# Current Perspectives on Treatment of Gram-Positive Infections in India: What Is the Way Forward?

**DOI:** 10.1155/2019/7601847

**Published:** 2019-04-07

**Authors:** Atul P. Kulkarni, Vasant C. Nagvekar, Balaji Veeraraghavan, Anup R. Warrier, Deepak TS, Jaishid Ahdal, Rishi Jain

**Affiliations:** ^1^Division of Critical Care Medicine, Tata Memorial Hospital, Homi Bhabha National Institute, Mumbai, Maharashtra 400012, India; ^2^Lilavati Hospital and Research Centre, Bandra Reclamation Rd, Bandra (West), Mumbai, Maharashtra 400050, India; ^3^Department of Clinical Microbiology, Christian Medical College & Hospital, Ida Scudder Road, Vellore, Tamil Nadu 632004, India; ^4^Aster Medicity, Kuttisahib Road, Cheranalloor, South Chittoor, Kochi, Kerala 682027, India; ^5^Department of Emergency Medicine, MS Ramaiah Medical College, M S Ramaiah Nagar, Mathikere, Bengaluru, Karnataka 560054, India; ^6^Medical Affairs, Wockhardt Ltd., Wockhardt Towers, Bandra Kurla Complex, Mumbai, Maharashtra 400051, India

## Abstract

The emerging antimicrobial resistance leading to gram-positive infections (GPIs) is one of the major public health threats worldwide. GPIs caused by multidrug resistant bacteria can result in increased morbidity and mortality rates along with escalated treatment cost and hospitalisation stay. In India, GPIs, particularly methicillin-resistant* Staphylococcus aureus* (MRSA) prevalence among invasive* S. aureus* isolates, have been reported to increase exponentially from 29% in 2009 to 47% in 2014. Apart from MRSA, rising prevalence of vancomycin-resistant enterococci (VRE), which ranges from 1 to 9% in India, has raised concerns. Moreover, the overall mortality rate among patients with multidrug resistant GPIs in India is reported to be 10.8% and in ICU settings, the mortality rate is as high as 16%. Another challenge is the spectrum of adverse effects related to the safety and tolerability profile of the currently available drugs used against GPIs which further makes the management and treatment of these multidrug resistant organisms a complex task. Judicious prescription of antimicrobial agents, implementation of antibiotic stewardship programmes, and antibiotic policies in hospitals are essential to reduce the problem of drug-resistant infections in India. The most important step is development of newer antimicrobial agents with novel mechanisms of action and favourable pharmacokinetic profile. This review provides a synopsis about the current burden, treatment options, and the challenges faced by the clinicians in the management of GPIs such as* MRSA*, Quinolone-resistant* Staphylococcus, *VRE, and drug-resistant pneumococcus in India.

## 1. Introduction

Ever since the introduction of penicillin in 1940s, the fight against infections by gram positive bacteria has seen many ups and downs. Gram-positive infections (GPIs) can result in a wide variety of diseases, including skin and soft tissue infections, surgical and trauma wound infections, urinary tract infections, gastrointestinal tract infections, pneumonia, osteomyelitis, endocarditis, thrombophlebitis, mastitis, meningitis, toxic shock syndrome, septicaemia, and infections of indwelling medical devices [1]. Today, among various pathogenic gram-positive bacteria,* Staphylococcus aureus, Streptococcus pneumoniae,* and enterococci stand out as being responsible for global resistance challenges, significant public health burden, and cost to healthcare [2]. Multidrug resistance among gram positive bacteria, especially methicillin-resistant* Staphylococcus aureus* (MRSA), has been a major healthcare concern worldwide [3].

Even in India, GPIs are a significant public health concern. Recent reports suggest that the prevalence of infections by multidrug resistant gram-positive bacteria is on the rise, with MRSA among invasive* S. aureus* isolates estimated to be 29% in 2009, which increased to around 47% in 2014 [4]. Further, in addition to delayed recuperation and long term disability, GPIs, especially MRSA infections, are associated with high mortality rates in India [5]. A 2010 study from Ahmedabad reported a clear trend in the emergence of gram positive bacteria in blood stream infections with* Staphylococcus* constituting up to 27.4% of all blood culture specimen isolates [6]. Methicillin-resistant* Staphylococcus* is considered to be endemic in India [7]. A direct consequence of this emergence of multidrug resistance (MDR) among gram-positive bacteria is the progressive depletion of effective antimicrobial agents to treat patients suffering from these infections. Therefore, MDR leads to increased mortality and morbidity, ICU admissions, complications, requirement of therapy by multiple antimicrobial agents, and increased over-all length of stay in the hospital. All these factors result in an overall increase in the treatment cost, which can be disastrous in a country like India where most of the healthcare expenditure is borne out-of-pocket [8, 9].

Commonly used antibiotics in India for treating various GPIs include beta lactams, vancomycin, macrolides, linezolid, fluoroquinolones, and doxycycline [5]. The objective of this review is to provide an overview about the current burden of GPIs, available treatment options, and challenges being faced by clinicians in managing GPIs in India.

## 2. Susceptible, Intermediate, and Resistant Bacteria

The antimicrobial sensitivity reporting by microbiology laboratories generally falls into three categories: susceptible, intermediate, and resistant [10].

A “susceptible” bacterial strain is one, whose in vitro growth is inhibited by an antibiotic, and there is a high likelihood of therapeutic success when the antibiotic is administered to patients in similar concentrations; methicillin-susceptible* S. aureus* (MSSA) is an example. An “intermediate” bacterial strain is one whose in vitro growth is inhibited by an antibiotic in such a concentration which, when used therapeutically, results in an uncertain therapeutic effect; examples include vancomycin-intermediate* S. aureus* (VISA), heterogeneous VISA (hVISA), and glycopeptide-intermediate* S. aureus *(GISA). A “resistant” bacterial strain is one whose in vitro growth is inhibited by an antibiotic in a concentration which, when used therapeutically, has a high likelihood of therapeutic failure; examples include MRSA, vancomycin-resistant* S. aureus* (VRSA), and vancomycin-resistant enterococci (VRE) [10].

## 3. Common GPIs in India and Available Treatment Options

### 3.1. Methicillin-Susceptible S. aureus (MSSA)

First generation cephalosporins such as cefazolin, and semisynthetic antistaphylococcal penicillins such as cloxacillin are considered the optimal antimicrobials for the definitive treatment of MSSA bacteremia [11]. Vancomycin can be initiated as an empirical therapy if the methicillin resistant pattern of the suspected* S. aureus* infection is unknown. If the isolate is methicillin sensitive, then, within 3 days, a definitive therapy with cloxacillin or cefazolin should be initiated [12, 13].

### 3.2. Methicillin Resistant S. aureus (MRSA)

First identified in the 1960s, MRSA is now a common cause of serious hospital-acquired infections. MRSA can be clinically grouped as community-associated (CA-MRSA) or hospital/healthcare-associated (HA-MRSA). Traditionally, it was thought that CA-MRSA causes skin and soft tissue infections (SSTIs), and HA-MRSA causes infections upon prolonged hospitalisation, in patients with indwelling devices or in patients undergoing dialysis or receiving immunosuppressive therapy. However, it is increasingly being observed worldwide, including in India, that CA-MRSA is gradually resembling HA-MRSA in being more invasive and transmissible than before [14]. In India, high rates of MRSA have been reported in clinical isolates from various studies, with rates as high as 54.8% (ranging between 32% and 80% among the* S. aureus* pool) [4].

Vancomycin remains the most important drug for the treatment of MRSA [15]. In addition, the infectious disease society of America (IDSA) considers daptomycin to be another useful drug for invasive MRSA infections [16]. Telavancin, ceftaroline, and linezolid may be used for second-line therapy of MRSA [17]. The 2016 Indian infectious disease treatment guidelines recommend the glycopeptides vancomycin and teicoplanin as drugs of first choice for MRSA, linezolid for MRSA-induced SSTIs, and daptomycin for complicated SSTIs and bacteremia due to MRSA [5]. Ceftobiprole is a new broad-spectrum, a “5th generation” cephalosporin which has activity against gram-positive bacteria, including MRSA [18].

There have been reports from different parts of India isolating MRSA strains with additional resistance to linezolid (Linezolid-resistant MRSA, LR-MRSA) [19] and to multiple antibiotics such as vancomycin, linezolid, and tigecycline (multidrug resistant* S. aureus*, MDRSA) [20]. The emergence of multiple drug resistance among* Staphylococcus aureus *isolates may be significant, though the exact prevalence and clinical implications remain to be known.

### 3.3. Vancomycin Resistance in MRSA

Vancomycin, which is a glycopeptide active against many gram-positive organisms, was first used in 1958 to treat infection by gram positive bacteria, including* S. aureus *resistant to methicillin [21].* S. aureus* strains with reduced susceptibility to vancomycin were isolated after nearly 40 years in 1996 in Japan and were termed vancomycin-intermediate* S. aureus* (VISA) [22].

An intermediate phenotype known as heterogeneous VISA (hVISA) frequently precedes the development of the VISA phenotype. A colony of hVISA is composed of a mixed cell population, in which most cells are susceptible to vancomycin (MIC ≤ 2 *μ*g/ml), and a small proportion of cells has vancomycin resistance, with an elevated MIC value (MIC ≥ 4 *μ*g/ml) [23]. It is now thought that VISA develops when hVISA infection is treated with glycopeptide antibiotics over prolonged time periods [24].

Many of the VISA strains also showed reduced susceptibility to the other glycopeptide antibiotic teicoplanin and were termed glycopeptide-intermediate* S. aureus *(GISA) [25]. Subsequently,* S. aureus* strains which were resistant to vancomycin (due to acquisition of* vanA* gene) were reported in the USA in 2002 and were termed as vancomycin-resistant *S. aureus* (VRSA) [21]. The first report of VISA/ VRSA from India was published in 2006 [26].

Treatment of VISA and VRSA can be challenging. For the treatment of infections by VISA strains with an MIC greater than 8 *μ*g/ml, usage of glycopeptides is generally not considered to be optimal [24, 27]. The IDSA guidelines recommend the usage of vancomycin alternatives such as a combination of high-dose daptomycin with another antibiotic including gentamicin, rifampicin, linezolid, trimethoprim-sulfamethoxazole (TMP-SMX), or a *β*-lactam, for the treatment of VISA and VRSA infections [16]. However, given the high prevalence of tuberculosis in India, it is generally not advisable to use rifampicin for this indication. If there is reduced susceptibility to daptomycin as well as in the VRSA/VISA strain, then the treatment options, according to the IDSA guidelines, include a combination or monotherapy with single use of quinupristin-dalfopristin, TMP-SMX, linezolid, or telavancin [16].

### 3.4. Vancomycin-Resistant Enterococci (VRE)

While* S. aureus* is pathogenic bacteria, enterococci are normal components of the human bowel flora. In healthy people, enterococci are not considered as primary pathogens and may occasionally cause urinary tract infections. However, enterococci have been known to cause opportunistic infections in hospitalised patients, and frequently such infections are resistant to multiple drugs including vancomycin [2]. Most opportunistic enterococcal infections are caused by* E. faecalis; *however,* E. faecium* infections, by virtue of a higher prevalence of multidrug resistance, are more problematic [2]. Apart from urinary tract infections, enterococci may cause bacteraemia, infective endocarditis, intra-abdominal and pelvic infections, infections of the CNS, and SSTIs [28].

*E. faecium* strains resistant to vancomycin were first isolated in England and France in 1986 [29] and were dubbed as VRE. In 1987, vancomycin-resistant strains of* E*. faecalis were reported in the United States [30]. In India, the first report of VRE was published from New Delhi, in 1999 [31]. The prevalence of VRE in India is increasing according to recent reports [32].

The optimal management of VRE depends upon the site of infection, species, and in vitro susceptibility of VRE to antibiotics. Linezolid has excellent activity against both* E. faecalis *and* E. faecium *and is the preferred drug for the treatment of VRE infections [28]. The 2016 Indian infectious disease treatment guidelines recommend high-dose ampicillin trial for VRE strains which retain susceptibility to ampicillin, daptomycin monotherapy in serious VRE infections, nitrofurantoin, and fosfomycin for UTI caused by susceptible VRE strains, and doxycycline, chloramphenicol, gentamicin, and streptomycin as part of combination chemotherapy during serious infections by susceptible strains [5]. VRE strains which are resistant to linezolid have also been reported; fortunately, so far this is rare and parallels increasing linezolid consumption [33].

### 3.5. Antibiotic Resistance among Streptococcus Pneumoniae

The first penicillin-resistant isolate of* Streptococcus pneumoniae* was reported in 1967 [34]. This led to the increasing use of macrolides for the treatment of pneumococcal infections. However, the wide-spread macrolide use resulted in an increased macrolide resistance in* S. pneumoniae*. Continued macrolide use is contributing to an expansion of macrolide-resistant* S. pneumoniae *[35]. The prevalence of macrolide resistance among* S. pneumoniae *is variable according to geography and is reported to constitute a wide range of < 10% to > 90% of all isolates [35]. The prevalence of macrolide resistance in pneumococci in 2015 in India was 32% (19% to 47%) [36]. Subsequently* Streptococcus pneumoniae* strains resistant to multiple antibiotics such as penicillin, tetracycline, erythromycin, chloramphenicol, rifampicin, and clindamycin were isolated in 1977 [37]. A recently published systematic review concluded that resistance of* S. pneumoniae* is highly prevalent with beta-lactams (penicillins: 13.8-41.8%, cephalosporins: <1-29.9%), macrolides (20-40%), clindamycin (21.8%), TMP-SMX (25-45%), and tetracyclines (25.9%), whereas resistance against fluoroquinolones (<1-2%) is low but increasing [38]. The prevalence of drug-resistant* S. pneumoniae* (DRSP) has also been reported to be increasing in India [39].

The development of multidrug resistance is compromising the management of pneumococcal infections. However, the clinical impact of the current levels of antibiotic resistance is not fully clear. Meningitis due to DRSP has poorer outcomes, but there is a considerable debate about the outcome of pneumonia due to DRSP [40]. The 2007 IDSA/ATS guidelines recommend that respiratory fluoroquinolones (moxifloxacin, gemifloxacin, or levofloxacin), or a beta-lactam alone or in combination with a beta-lactamase inhibitor (high dose amoxicillin or amoxicillin-clavulanate) along with doxycycline are to be used in areas with >25% infection rate with “high-level” macrolide-resistant* S. pneumoniae *[41].

### 3.6. Quinolone Resistance in Gram Positive Bacteria

Quinolones have become a vital part of antimicrobial drugs against a large variety of both gram-positive and gram-negative bacteria in the present day. However, quinolone resistance is increasingly being observed nowadays and is threatening the use of this useful group of drugs [42]. In a 2013 study, resistance to ciprofloxacin was observed in up to 57.6% of* Staphylococcus aureus* isolates, and 37.6% of the isolates were resistant to all fluoroquinolones. Further, among the MRSA strains, resistance to fluoroquinolones was quite high: 92.5% of the isolates were resistant to ciprofloxacin, and 80.4% of the isolates were resistant to ofloxacin. Only 7.5% of the MRSA were sensitive to all 6 fluoroquinolones tested in the study [43]. While fluoroquinolone resistance is deemed to be < 2% in* Streptococcus pneumoniae* [44], a 2008 study reported that when fluoroquinolones were used to treat tuberculosis in children, there was an emergence of invasive pneumococcal disease which was nonsensitive to levofloxacin and was associated with a high mortality rate (45%) [45].

A summary of recommended treatment options for important GPIs is given in Table 1.

## 4. GPIs in ICU Setting

Patients admitted in intensive care units (ICUs) are at a high risk of developing infections. Studies from different regions of India have reported that the prevalence of both primary and secondary infections is quite high with both gram-positive and gram-negative bacteria. While the gram-negative bacteria predominate the ICU infections in India, the drug-resistant gram-positive organisms (especially MRSA and VRE) also cause significant amount of ICU-related infections. In a 2015 study, it was reported that* Staphylococcus aureus *and* Enterococcus* species were responsible for 8.2% and 5.0% of ICU infections, respectively [46]. Another study from 2016 reported that GPIs accounted for 15.9% of ICU infections, second to gram-negative organisms at 68.9% [47].

When blood stream infections (BSIs) in ICUs are taken into consideration, the most commonly isolated organisms are gram-positive bacteria, mainly* Staphylococcus aureus* [48, 49]. MRSA is also an important organism causing BSIs: in the recently reported EUROBACT study involving 1,156 patients admitted to ICUs with a diagnosis of BSIs, MRSA was isolated in up to 50% cultures [50]. Further, isolation of* S. aureus* in blood cultures is independently associated with increased mortality in ICU-BSI patients [51]. A recent study reported that the overall mortality rate among patients with multidrug resistant GPIs was 10.8%; however, in ICU settings, the mortality rate was as high as 16% [52].

## 5. Challenges and Way Forward

Gram-positive bacteria are responsible for a diverse range of diseases, ranging from mild SSTIs to severe infections such as meningitis and subsequent life-threatening sepsis. The emergence of antimicrobial resistance among the gram-positive bacteria has resulted in a large reduction in available options of antimicrobial drugs, thereby presenting a challenge to clinicians in treating serious GPIs, such as pneumonia, sepsis, and meningitis. The direct outcome of the emergence of antibiotic resistance is an increased healthcare cost [53].

Increased use and misuse of antibiotics are among the foremost contributors for the development of antibiotic resistance, especially in India. In fact, as reported in 2014, India was the largest consumer of antibiotics for human health in the world at 12.9 × 10^9^ units, followed by China and the US at 10.0 × 10^9^ units and 6.8 × 10^9^ units, respectively [54]. In the Indian setting, a combination of factors such as poor public health systems, high rates of infectious disease, inexpensive antibiotics, rising incomes, and increasing prevalence of resistant pathogens is responsible for the increasing burden of difficult-to-treat infections [4, 55].

Another problem with the currently available drugs is their spectrum of adverse effects. A list of the most common serious adverse effects of the currently available antibiotics active against resistant gram-positive bacteria is presented in Table 2.

A major programme towards reducing the unnecessary and indiscriminate usage of high-end antibiotics is the implementation of antibiotic policy in each hospital and the initiation of antibiotic stewardship programmes (ASP). It is recommended that each healthcare institution should develop its own ASP, and it should be based on both international and national guidelines, as well as the local epidemiological and microbiological data. This is necessary to optimize the usage of antimicrobials among hospitalized patients, which would in turn improve the patient outcomes, reduce the unwanted outcomes of antimicrobial use, and thus ensure a cost-effective therapy [56]. Other steps for combating resistance include routine usage of antimicrobial susceptibility testing using novel techniques (techniques involving PCR, mass spectrometry, microarrays, microfluidics, flow cytometry, etc.), periodic antibiotic monitoring and supervision, development, and continuing medical education programmes for clinicians. Improvement in hygiene and reduction in the veterinary usage of antibiotics may also have a significant role in this direction [57]. A more difficult option would be to control and regulate the nonprescription sales of antimicrobials and to curb the problem of substandard and illegitimate antimicrobials in India [58].

## 6. Newer Antibiotics for GPIs

Probably the most important step to strengthen our existing armamentarium of antibiotics against GPIs would be to develop new antibiotics [59]. It is interesting to note that most of the groups of antibiotics being used were discovered prior to 1960. Only two new groups of systemic antibiotics were introduced to the market during the past two decades: linezolid and related oxazolidinones in 2000, and daptomycin and related lipopeptides in 2003 [57, 60]. Newer antibiotics belonging to established classes, such as delafloxacin, levonadifloxacin and its L-alanine ester prodrug alalevonadifloxacin, solithromycin, omadacycline, and some drugs with novel targets such as inhibitors of peptidyl deformylase and fatty acid synthase are in the pipeline for the treatment of gram-positive infections [61–64]. A brief summary of some newer antibiotics has been provided in Table 3.

Thus, the need of the hour is the development of new antimicrobial agents that act by novel mechanisms and have good antimicrobial spectrum, with favourable pharmacokinetic and pharmacodynamic properties.

## 7. Conclusion

Gram positive bacteria are responsible for causing significant infections in the healthcare and ICU setting. The development of drug resistance in these organisms is a serious problem, leading to difficult-to-treat infections by MRSA, VISA, VRSA, VRE, and other multidrug resistant organisms. The prevalence of resistance is bound to increase with increased irrational use of antibiotics. Steps such as restricting usage of antibiotics and antibiotic stewardship programmes need to be enforced strictly. The most important step, however, is the increased push towards development of newer antibiotics against the gram-positive bacteria with novel targets.

## Figures and Tables

**Table 1:** Gram positive organisms and recommended antimicrobial agents.

Organism	Recommended drugs	References
MSSA	Cefazolin, Cloxacillin	[11]
MRSA	Vancomycin, Daptomycin, Teicoplanin, Linezolid, Ceftaroline, Tigecycline	[15–17]
VISA, hVISA, VRSA	Combination of high-dose daptomycin with another antibiotic including gentamicin, rifampicin, linezolid, trimethoprim-sulfamethoxazole (TMP-SMX), or a *β*-lactam	[16]
VRE	Linezolid, High-dose ampicillin, daptomycin; nitrofurantoin, Fosfomycin (UTI); doxycycline, chloramphenicol, gentamicin, streptomycin (combination therapy)	[5, 28]
DRSP	Respiratory fluoroquinolones (moxifloxacin, Gemifloxacin, or levofloxacin), or a beta-lactam alone or in combination with a beta-lactamase inhibitor (high dose amoxicillin or amoxicillin-clavulanate) along with doxycycline	[41]

**Table 2:** Significant adverse effects seen with currently used antibiotics.

Antibiotic	Significant Adverse Effects	Reference
Vancomycin	Nephrotoxicity, hypotension, hypersensitivity reactions; Red man syndrome	[65]
Linezolid	Thrombocytopenia, optic neuropathy, peripheral neuropathy, lactic acidosis	[66]
Daptomycin	Myopathy, rhabdomyolysis, eosinophilic pneumonia, anaphylactic hypersensitivity reactions	[67]
Tigecycline	Bone growth inhibition, teratogenicity, hepatic toxicity, elevated liver enzymes	[68]
Clindamycin	Pseudomembranous colitis, thrombophlebitis, azotemia, agranulocytosis	[69]

**Table 3:** Some new drugs recently approved and in pipeline for treatment of gram-positive infections.

Name of the drug	Class	Mechanism of action	Important GP organisms covered^*∗*^	Dose and Route^†^	Phase of testing^‡^	Serious Adverse effects*∗*^‡^
Delafloxacin	Fluoro-quinolone	Inhibit DNA gyrase and Topoisomerase IV	MRSA, MSSA, *St. pyogenes*, *E. faecalis*	300 mg IV/ 450 mg oral	USFDA approved in June 2017	Tendinitis and tendon rupture, peripheral neuropathy, Central nervous system effects
Nemonoxacin			MRSA, VRSA	250-750 mg, oral, IV	Phase III	None reported
Levonadifloxacin			MRSA, VRSA	1000 mg oral, 800 mg IV	Phase III	None reported
Alalevonadifloxacin			MRSA, VRSA	1000 mg oral	Phase III	None reported

Solithromycin	Fluoro-ketolide	Inhibits protein synthesis by binding to 50s ribosome subunit and blocking peptide chain elongation	*S. aureus, St. pneumoniae*	800 mg oral	Phase III	Possible hepatotoxicity

Omadacycline	Tetracycline	Inhibits protein synthesis by binding to 30s ribosome subunit and blocking binding of aminoacid-tRNA with mRNA	*S. aureus (MSSA), St. pneumoniae*	100-200 mg IV; 300-450 mg oral	USFDA approved in October 2018	Similar to other tetracyclines
Eravacycline			*E faecalis, E faecium, S. Aureus, St anginosus *group	1 mg/kg IV infusion	USFDA approved in August 2018	Similar to other tetracyclines

Tedizolid	Oxazolidinone	Inhibits protein synthesis by binding to the 50S ribosome subunit	MRSA, MSSA, *St. pyogenes, St. agalactiae, S. anginosus, E. faecalis*	200 mg oral or IV infusion	USFDA approved in June 2014	Similar to linezolid

Lanopepden (GSK'322)	Peptidyl deformylase inhibitor	Inhibits bacterial protein synthesis	*St. pneumoniae*, *St. pyogenes*, *S. aureus*	1500 mg oral	Phase II	None reported

AFN-1252	Fabl (enoyl ACP reductase) inhibitor	Interferes with essential bacterial fatty acid biosynthetic pathway	*S. aureus, S. epidermidis,* other staphylococci	200 mg oral	Phase II	None reported

Note: ^*∗*^this list is not exhaustive; ^†^these are suggested doses; detailed dosing and regimens may vary according to indication; ^**‡**^as on December 2018.
